# Visit‐to‐visit office blood pressure variability revisited in SPRINT

**DOI:** 10.1111/jch.14313

**Published:** 2021-07-01

**Authors:** Michiaki Nagai, Kazuomi Kario

**Affiliations:** ^1^ Department of Cardiology Hiroshima City Asa Hospital Hiroshima Japan; ^2^ Division of Cardiovascular Medicine Department of Medicine, Jichi Medical University School of Medicine Tochigi Japan

**Keywords:** all‐cause mortality, blood pressure variability, Framingham Risk Score

While hypertension is an important predictor of cardiovascular disease (CVD),^1^ blood pressure (BP) fluctuation is caused by complex interactions of external environmental stimuli and internal physical status. BP variations can emerge when subjects are observed over the course of repeated clinical visits. Visit‐to‐visit office BP variability (VVV) has been reported to predict cerebral infarction in Japanese elderly hypertensives.^2^ In a large cohort of patients with previous transient ischemic attack (TIA; the UK‐TIA Aspirin Trial), and a broad population of patients with hypertension in the Anglo‐Scandinavian Cardiac Outcomes Trial Blood Pressure Lowering Arm (ASCOT‐BPLA), VVV in systolic BP (SBP) was shown to be a strong predictor of stroke independent of average SBP.^3^ The increased VVV SBP variability was associated with CVD mortality.^4^ However, the mechanisms by which increased VVV caused unfavorable prognosis remain poorly understood.

In an earlier analysis of the Systolic Blood Pressure Intervention Trial (SPRINT)^5^ that enrolled hypertensives with increased risks of CVD who were ≥50 years old and had SBP ≥130 mmHg, evidence of CVD, chronic kidney disease (CKD), or a 10‐year Framingham Risk Score (FRS) score ≥15%, VVV was not significantly associated with the primary composite end point of fatal and nonfatal CVD or with heart failure (HF) or stroke hospitalizations. In the primary analysis, although the highest quintile of VVV was associated with all‐cause mortality, the significant association was attenuated after adjusting for confounding factors.^5^

The paper by Cheng and coworkers^6^ in this issue of the *Journal* provides several new insights into the relationship of two VVV parameters (the variability independent of the mean [VIM] and the difference of maximum minus minimum [MMD]) with all‐cause mortality in the 7996 hypertensives from SPRINT.^6^

The first key finding is that VVV SBP was an independent predictor of all‐cause mortality after adjustment of conventional risk factors or FRS.^6^ While VVV SBP was defined as the coefficient of variation (CV) (standard deviation [SD] of average SBP divided by average SBP) in the earlier SPRINT analysis,^5^ VIM and MMD were used as indices of VVV in this SPRINT analysis reported by Cheng and coworkers.^6^ VIM SBP was found to reduce the tight correlation between CV SBP and average SBP, and MMD directly reflected the fluctuation of SBP. Thus, in comparison with CV SBP, both VIM and MMD have been shown to have stronger associations with all‐cause mortality even after adjusting for other conventional factors or FRS.^5^, ^6^

The second key finding was that VVV SBP and FRS were both significant risk factors for all‐cause mortality, and that higher VVV SBP combined with higher FRS conferred an increased risk for all‐cause mortality.^6^ Interestingly, that association was also found in the intensive‐therapy group that had an SBP target of less than 120 mmHg.^6^ These results indicate that hypertensives at a high‐risk of CVD are most vulnerable to BP fluctuation, even if SBP is strictly controlled, and provide a possible link for the pathophysiology underlying the relationship between VVV and poor prognosis in relation to atherosclerosis and arteriosclerosis, which might serve as an explanation for this phenomenon.^6^, ^7^

Wide oscillations in BP have been considered to increase the extent of oscillatory shear stress in the macrovascular system,^8^ and such increase in shear stress has been shown to cause a sustained activation of pro‐oxidant processes with increasing NADH oxidase activity^9^ and stimulation of adhesion molecule expression,^10^ resulting in redox‐sensitive gene expression. The alteration of vessel wall tension associated with the increased VVV might initiate atherosclerosis formation due to unique signals generated by oscillatory shear stress.^9^ ^.^ ^10^

While VVV was shown to be associated with artery remodeling in a cross‐sectional analysis,^11^ we reported the relationship between VVV and arterial stiffness of the common carotid artery in 164 elderly patients with one or more cardiovascular risks (79.7 years old at baseline, female 75%).^12^ During the mean follow‐up period of 4.2 years, VVV SBP had a significant positive association with the change of carotid stiffness parameter β.^12^ In the coronary artery risk development in young adults (CARDIA) study,^13^ the association between VVV SBP and 10‐year percent change in arterial stiffness was investigated among 1122 middle‐aged individuals without antihypertensive medications. In a multiple linear regression analysis, the group with the highest quintile of VVV SBP had a higher decline in the distensibility coefficient as well as a higher progression in Young's elastic modulus compared with those in the lowest quintile of VVV SBP independently of average SBP level.^13^

It is not completely clear whether the increased VVV is a cause or simply an index of increased arterial stiffness. One major determinant of BP variability is the sensitivity of baroreceptor function.^14^ Vascular structural changes may reduce baroreceptor sensitivity (BRS) in hypertension. Reduced large arterial compliance appears to contribute to the depressed BRS in young and middle‐aged hypertensive individuals.^15^ Stiffening itself might enhance BP fluctuations in association with minor changes in cardiac stroke volume. However, the inverse relationship between BP variability and BRS was suggested to be independent of the reduction in BRS accompanied with BP increase and aging.^8^, ^16^ Thus, larger prospective studies investigating the impact of arterial stiffness on VVV will be needed before concluding that a causal relation exists between them.

A recently proposed novel disease entity, systemic hemodynamic atherothrombotic syndrome (SHATS),^17^, ^18^ is characterized by a vicious cycle between hemodynamic stress and vascular disease, and is a risk factor for CVD and target organ damages (TODs). In the SPRINT,^6^ this concept would also apply for the relationship between VVV and prognosis specifically in the hypertensives with higher FRS (Figure 1). The novel contribution of SHATS is its synergistic consideration of VVV and hemodynamic stress in relation to vascular disease, because exaggerated VVV might be reciprocally associated with TODs involving the brain (cerebral small “strain” vessel disease,^19^ cerebral hypoperfusion,^20^ cognitive dysfunction,^21^ and Alzheimer's disease^22^, ^23^, ^24^, ^25^) and heart (coronary artery remodeling,^7^, ^26^ left ventricular remodeling,^25^ increased NT‐proBNP,^26^ and HF^27^).

**Figure 1:**
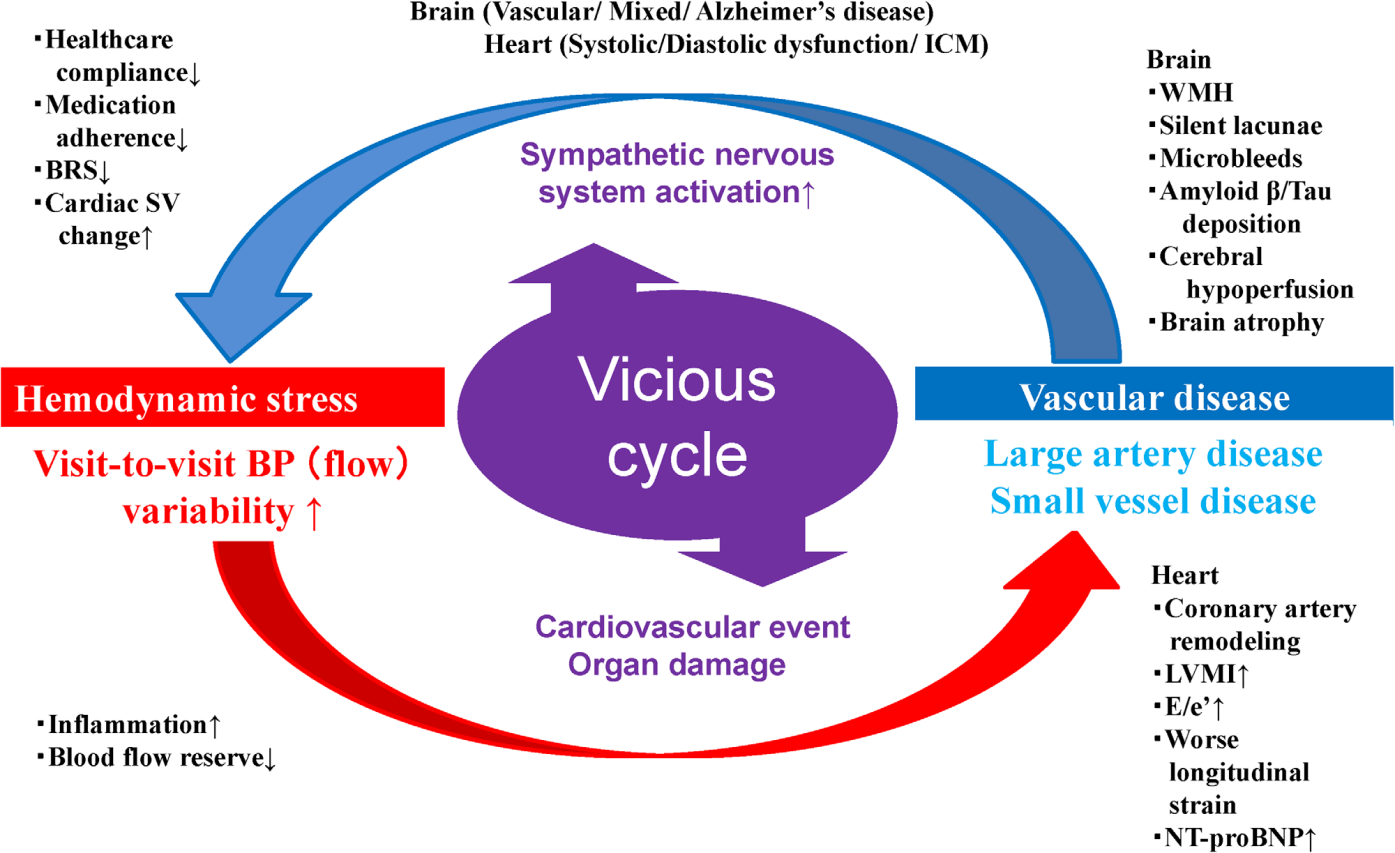
Application of systemic hemodynamic atherothrombotic syndrome (SHATS) to clarify the relationship between visit‐to‐visit BP variability and vascular disease: risk for cerebro‐cardio‐vascular disease is accelerated via a vicious cycle of hemodynamic stress and diseases in the brain and heart. BP indicates blood pressure; BRS, baroreceptor sensitivity; ICM, ischemic cardiomyopathy; LVMI, left ventricular mass index; NT‐proBNP, N‐terminal pro‐BNP; SV, stroke volume; WMH, white matter hyperintensity. (Revised from Kario,^17^ J Clin Hypertens 2019;21:1011‐1015.)

Until now, there have been few reports assessing the relationship between VVV and unfavorable outcome according to the severity of FRS. The data presented in the manuscript reported by Cheng and coworkers^5^ would thus make an important contribution, provided that they are considered within the context of the precise pathophysiology underlying that relationship.

## CONFLICT OF INTEREST

K. Kario has received research grants from Omron Healthcare and A&D Co. outside the submitted work. The other author declare no conflict of interest.
